# Supplementary figures and images for: Isolation and characterization of 24 phages infecting the plant growth-promoting rhizobacterium Klebsiella sp. M5al
Source: PLoS One. 2025 Feb 21;20(2):e0313947. doi: 10.1371/journal.pone.0313947 (PMC11845039; doi:10.1371/journal.pone.0313947)

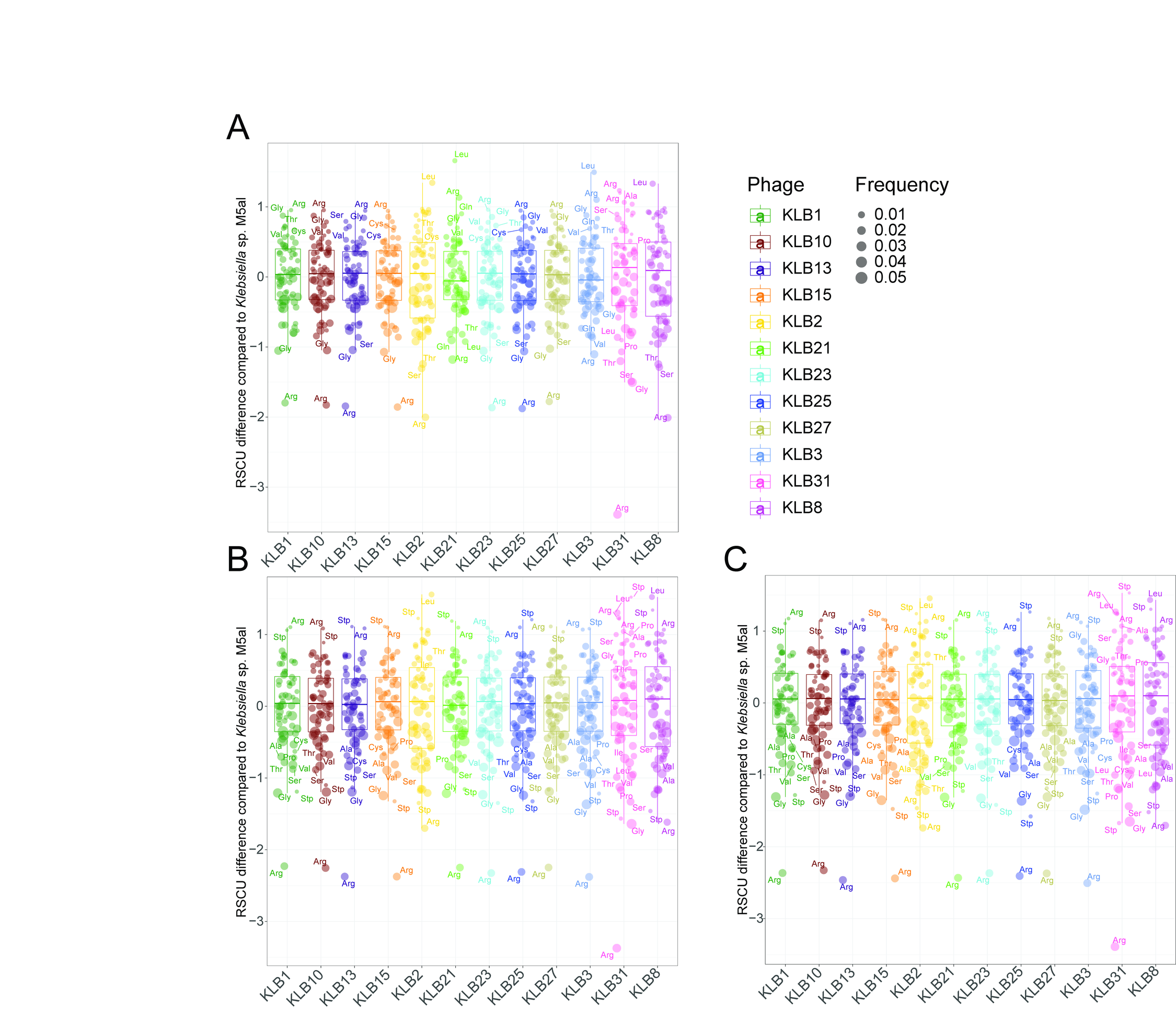

Supplement: S1 Fig — (A) cosine similarity of codons of each phage compared to Klebsiella sp. M5a1 for all genes, (B) genes in the late stage of phage infection, and (C) structural genes in the late stage of phage infection. (TIF) [file pone.0313947.s001.tif]

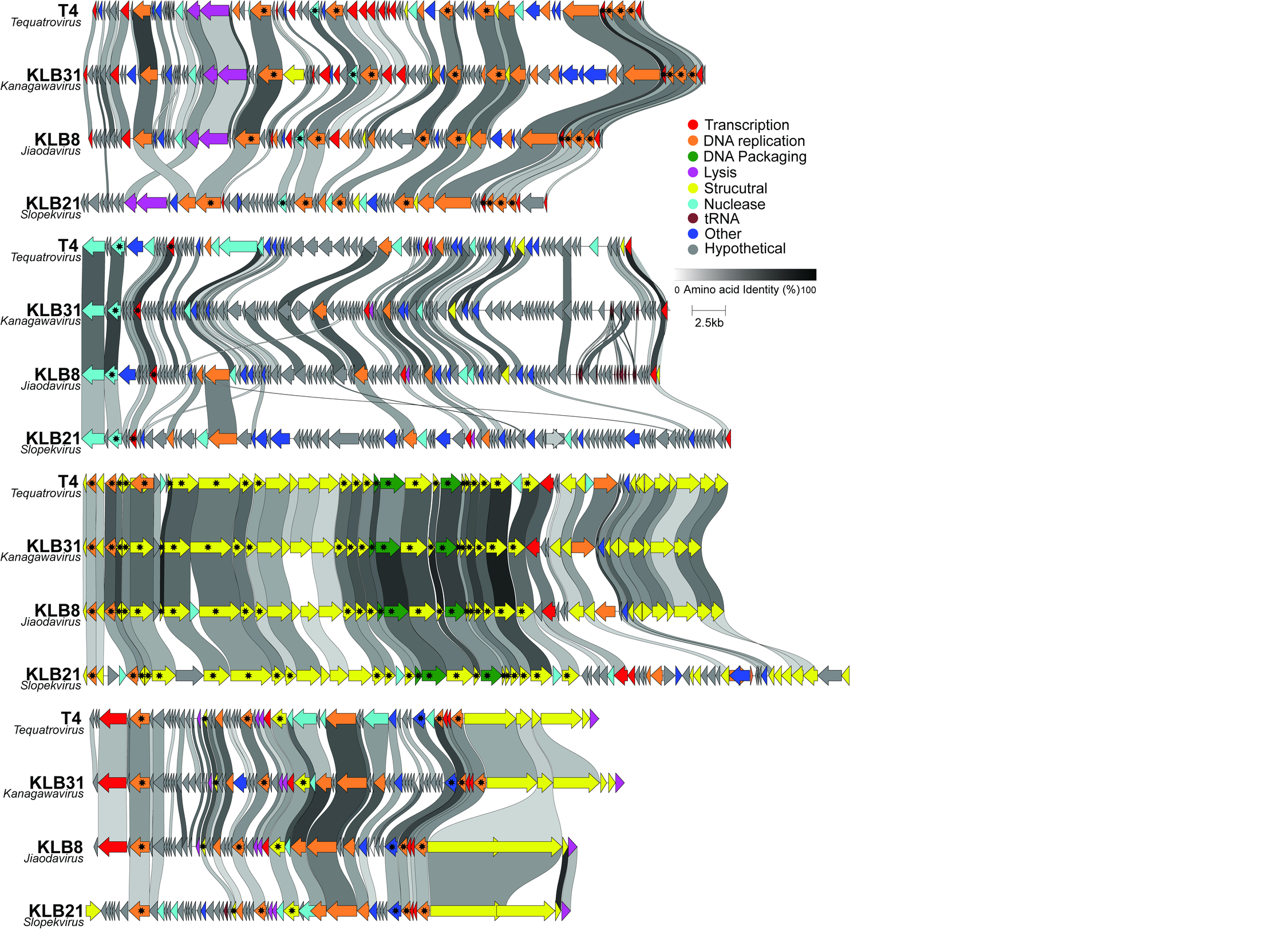

Supplement: S2 Fig — Arrows represent forward (right) or reverse (left) open reading frames (ORFs), ORFs are color-coded by function, and ORFs shared across genomes are connected by shading that denotes their percent identity. Core ORFs shared across all RefSeq genomes in this family are marked with a black star. The genome figure was made using Clinker. The genome was split into four sections to better zoom in on the individual genes. (TIF) [file pone.0313947.s002.tif]

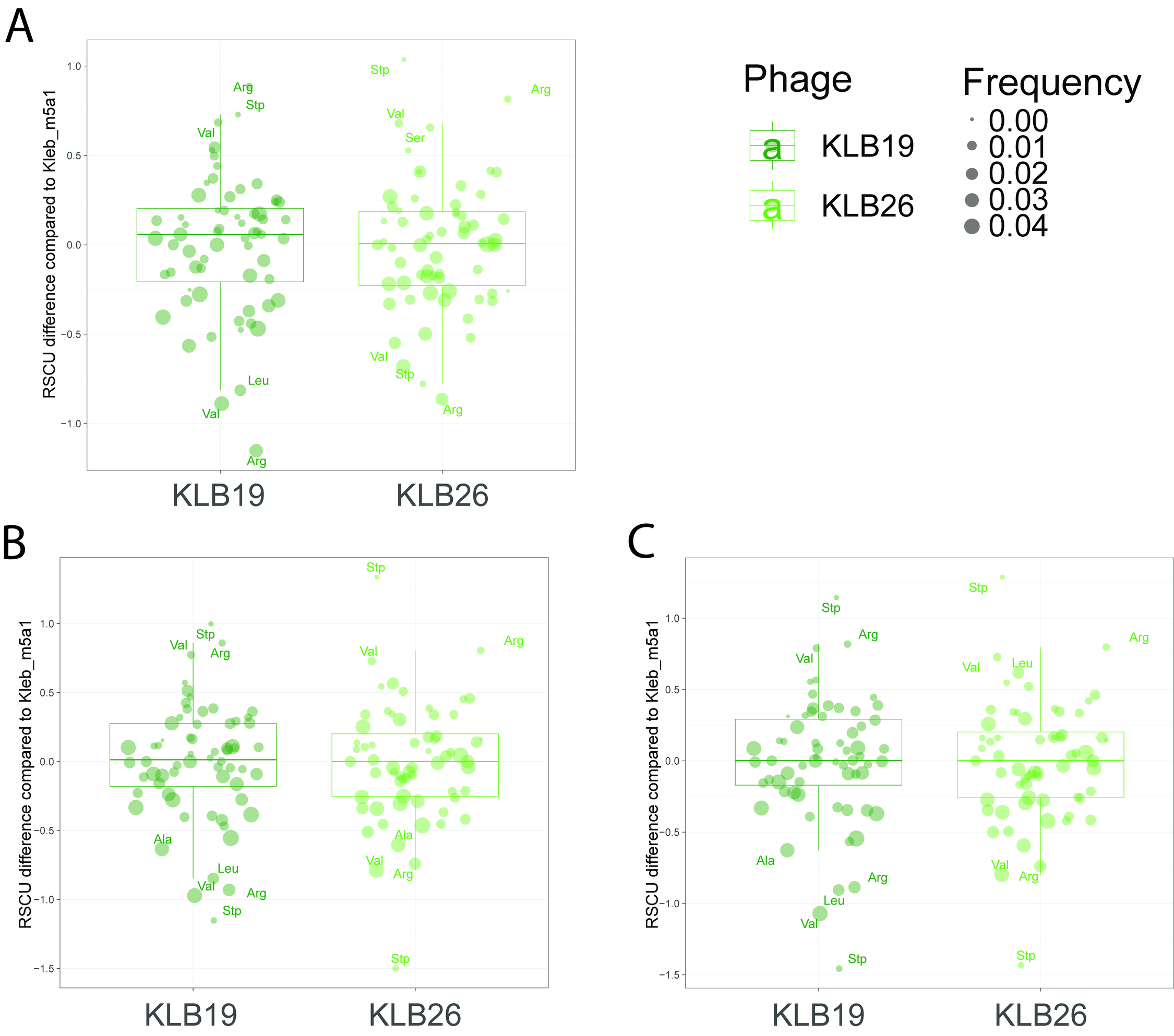

Supplement: S3 Fig — (A) Cosine similarity of codons of each phage compared to Klebsiella sp. M5a1 for all genes, (B) genes in the late stages of phage infection, and (C) structural genes in the late stage of phage infection. (TIF) [file pone.0313947.s003.tif]

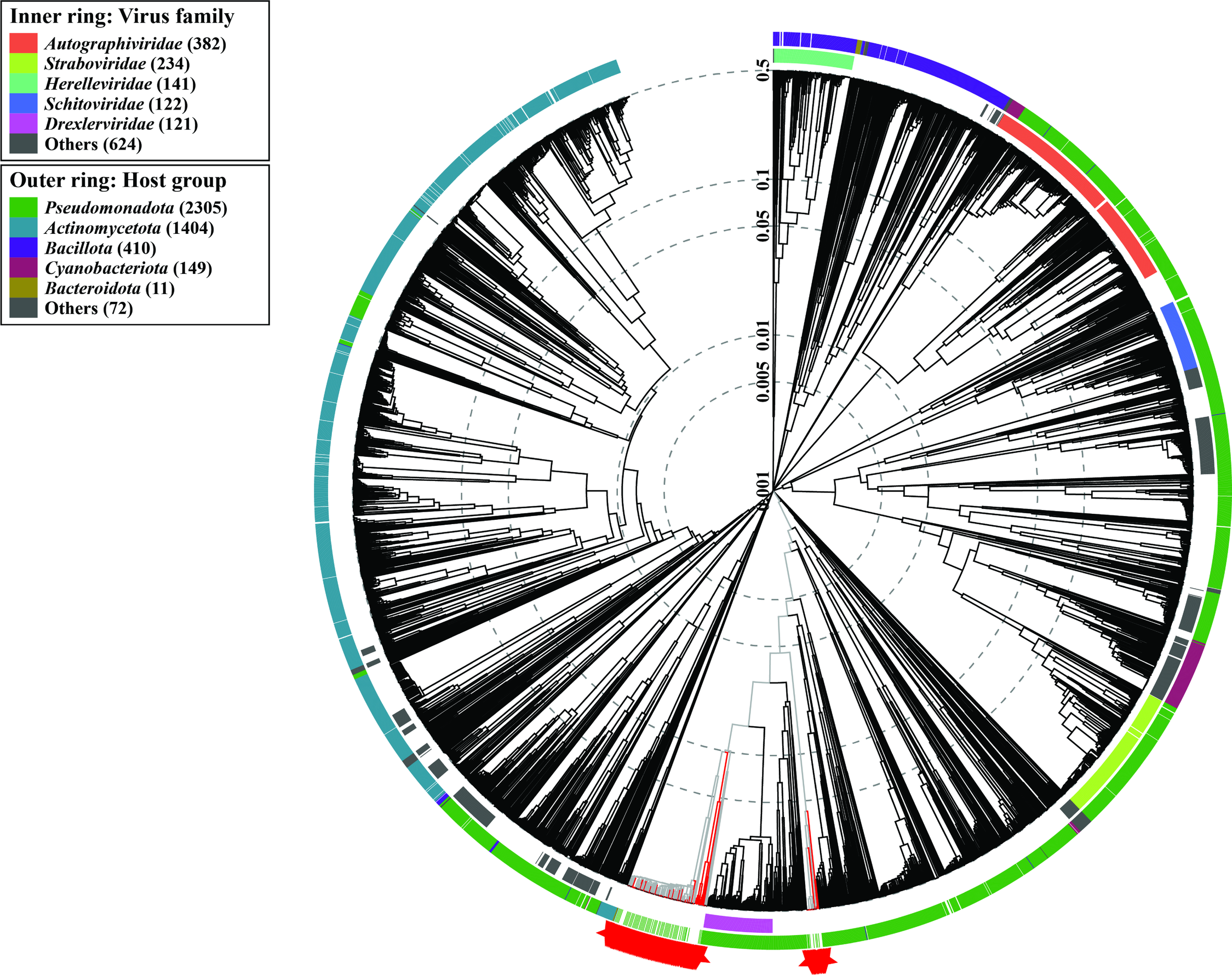

Supplement: S4 Fig — The red stars are the Ca. Mavericidae and Ca. Rivulusviridae phages. (TIF) [file pone.0313947.s004.tif]

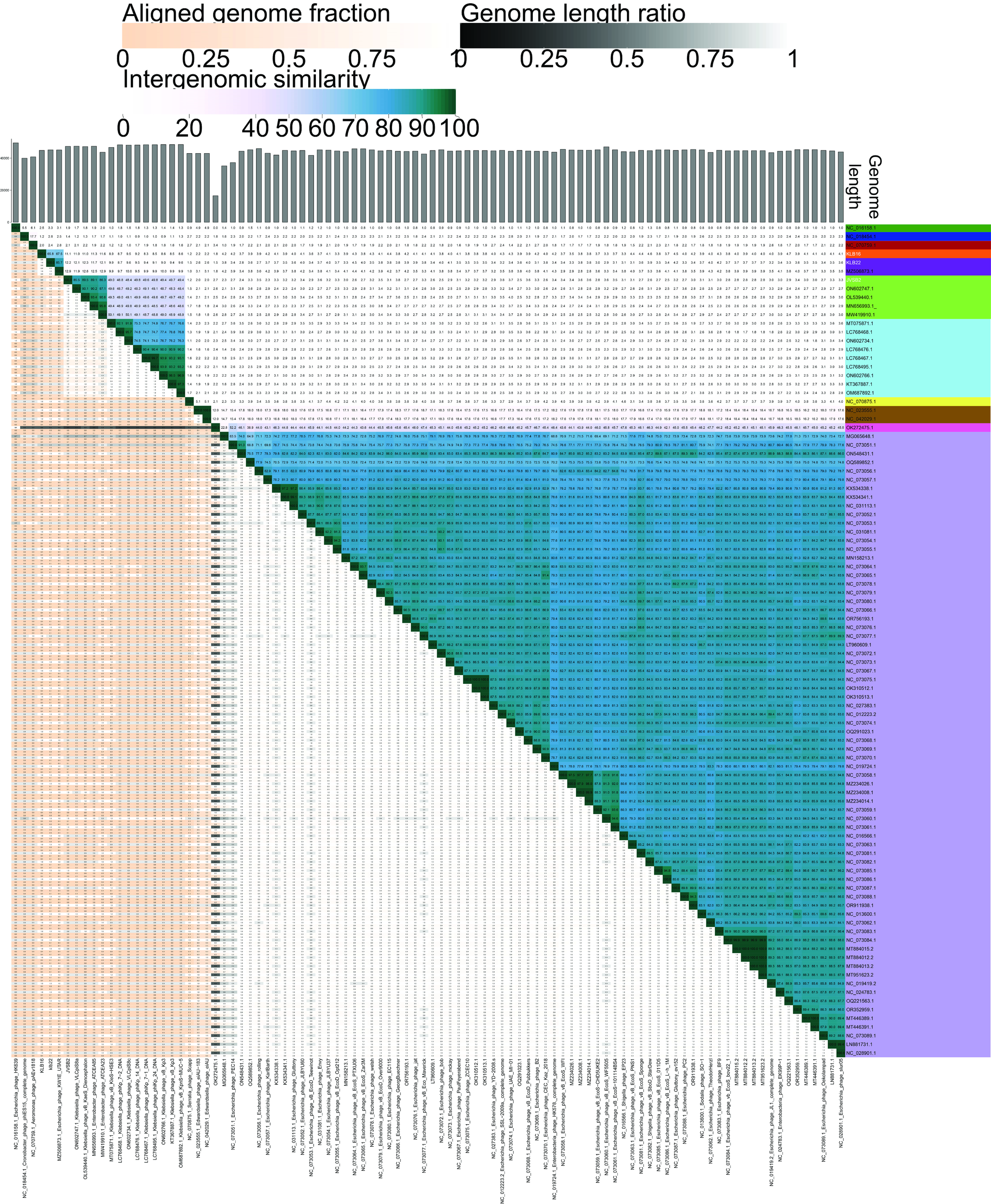

Supplement: S5 Fig — Phages with an intergenomic similarity ≥70% were grouped into a phage genus. Eleven phage genera of Mavericviridae are colored (green) Bowlingvirus, (dark blue) Gwanakvirus, (red) Yanchengvirus, (orange) Alumvirus, (purple) Buckeyevirus, (light green) Ashvirus, (light blue) Kijivirus, (yellow) Tamuvirus, (brown) Auburnvirus, (pink) Hildvirus, (light purple) Dhillonvirus. Phages labeled in white are the phages isolated in this study. (TIF) [file pone.0313947.s005.tif]

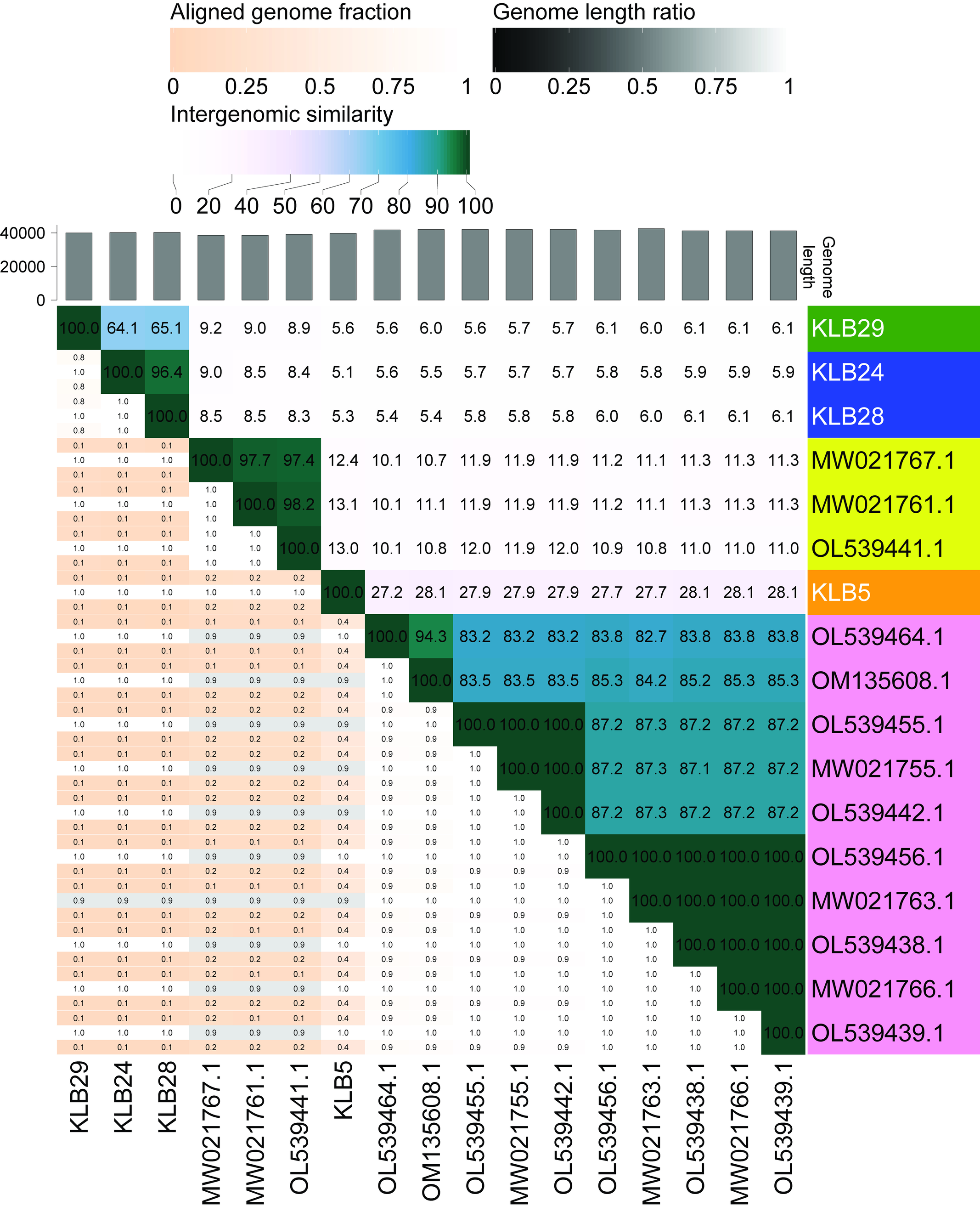

Supplement: S6 Fig — Phages with an intergenomic similarity ≥70% were grouped into a phage genus. 5 phage genera of Rivulusviridae are color-coded (green) Colbvirus, (blue) Sherbvirus, (yellow) Lucvirus, (orange) Darbyvirus, and (pink) Cinnavirus. Phages labeled in white are the phages isolated in this study. (TIF) [file pone.0313947.s006.tif]

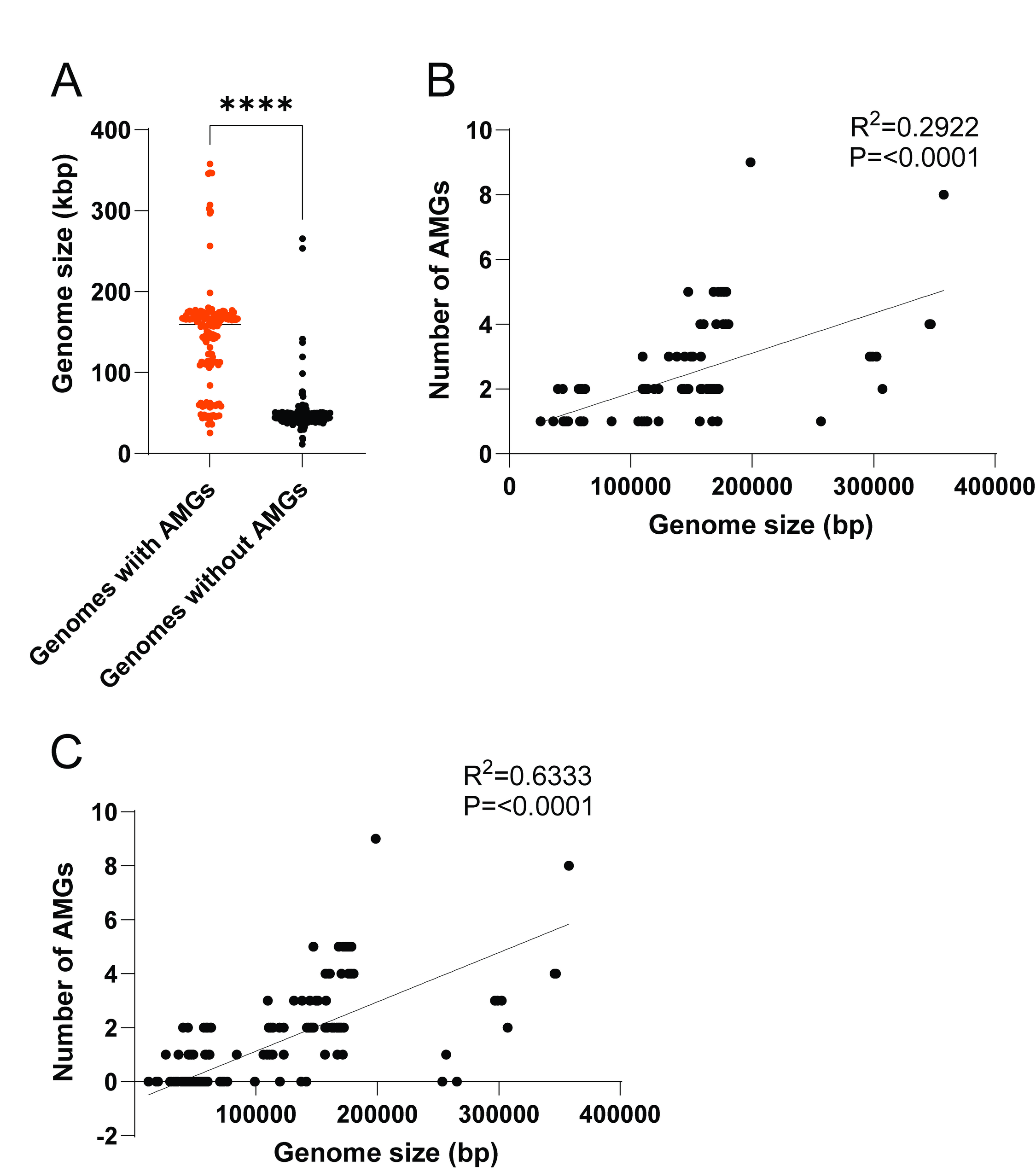

Supplement: S7 Fig — (A) Comparison of the genome sizes of phages that contain AMGs against phages that did not encode AMGs. A parametric, two-tailed, unpaired T-test was used to test statistical significance between the two groups. (B) Linear regression of phage containing AMGS compares genome size and the number of AMGs. (C) Linear regression of all Klebsiella phages genome sizes and number of AMGs. R2 and statistical significance were calculated using standard settings of linear regression in GraphPad. All figures were made in GraphPad. (TIF) [file pone.0313947.s007.tif]

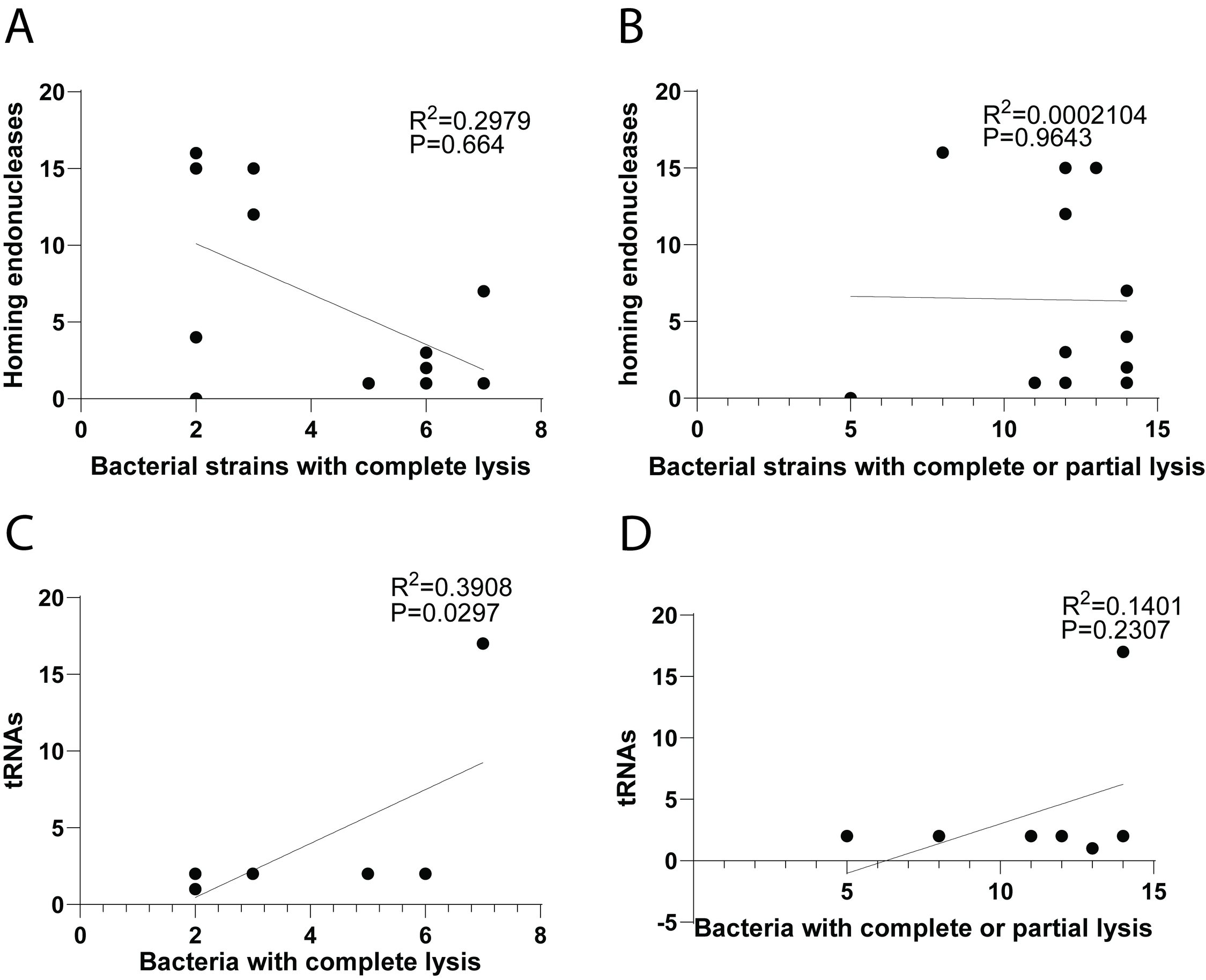

Supplement: S8 Fig — (A) Number of phage encoded homing endonucleases and number of bacteria a phage complete lyses and (B) completely or partially lyses. The number of phage-encoded tRNAs and the (C) number of bacteria a phage completely lyses and (D) completely or partially lyses. Linear regression of all Klebsiella phage genome sizes and number of AMGs. R2 and statistical significance were calculated using standard settings of linear regression in GraphPad. All figures were made in GraphPad. (TIF) [file pone.0313947.s008.tif]

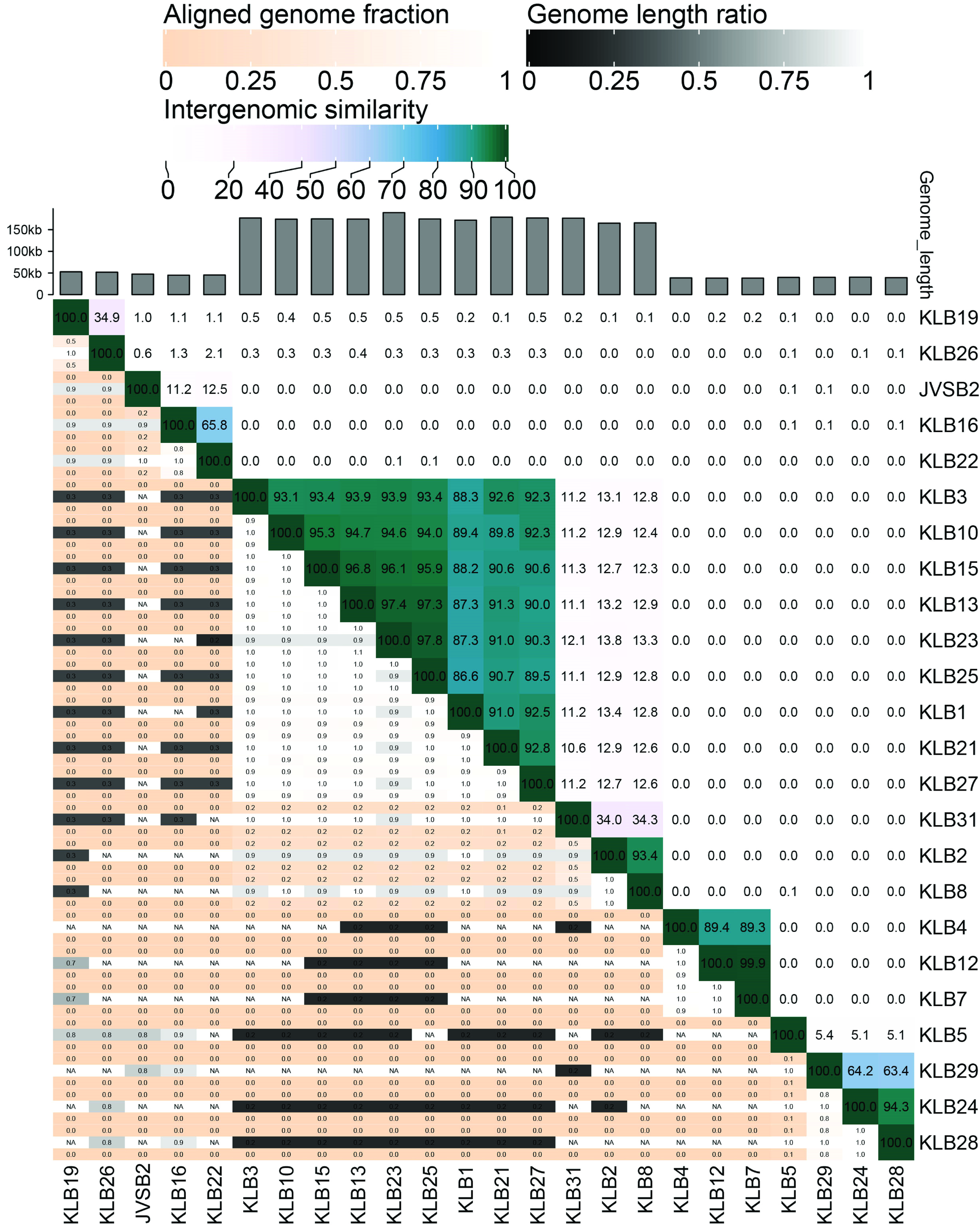

Supplement: S9 Fig — (TIF) [file pone.0313947.s009.tif]
